# The Broad Immunomodulatory Effects of IL-7 and Its Application In Vaccines

**DOI:** 10.3389/fimmu.2021.680442

**Published:** 2021-12-10

**Authors:** Juan Huang, Zhiyao Long, Renyong Jia, Mingshu Wang, Dekang Zhu, Mafeng Liu, Shun Chen, Xinxin Zhao, Qiao Yang, Ying Wu, Shaqiu Zhang, Bin Tian, Sai Mao, Xumin Ou, Di Sun, Qun Gao, Anchun Cheng

**Affiliations:** ^1^ Research Center of Avian Disease, College of Veterinary Medicine of Sichuan Agricultural University, Chengdu, China; ^2^ Institute of Preventive Veterinary Medicine, Sichuan Agricultural University, Chengdu, China; ^3^ Key Laboratory of Animal Disease and Human Health of Sichuan Province, Sichuan Agricultural University, Chengdu, China

**Keywords:** IL-7, biological function, mechanism, vaccine, molecular adjuvant

## Abstract

Interleukin-7 (IL-7) is produced by stromal cells, keratinocytes, and epithelial cells in host tissues or tumors and exerts a wide range of immune effects mediated by the IL-7 receptor (IL-7R). IL-7 is primarily involved in regulating the development of B cells, T cells, natural killer cells, and dendritic cells *via* the JAK-STAT, PI3K-Akt, and MAPK pathways. This cytokine participates in the early generation of lymphocyte subsets and maintain the survival of all lymphocyte subsets; in particular, IL-7 is essential for orchestrating the rearrangement of immunoglobulin genes and T-cell receptor genes in precursor B and T cells, respectively. In addition, IL-7 can aid the activation of immune cells in anti-virus and anti-tumor immunity and plays important roles in the restoration of immune function. These biological functions of IL-7 make it an important molecular adjuvant to improve vaccine efficacy as it can promote and extend systemic immune responses against pathogens by prolonging lymphocyte survival, enhancing effector cell activity, and increasing antigen-specific memory cell production. This review focuses on the biological function and mechanism of IL-7 and summarizes its contribution towards improved vaccine efficacy. We hope to provide a thorough overview of this cytokine and provide strategies for the development of the future vaccines.

## Introduction

In 1987, Hunt et al. discovered a cytokine that promoted the development of pro- and pre-B cells while exploring the potential role of bone marrow stromal cells in pre-B cell subset growth (1). The following year, Namen et al. isolated and purified a cytokine from the culture supernatant of mouse bone marrow stromal cells and named it lymphocyte growth factor-1 (lymphopoetin-1, LP-1) (2). This cytokine was officially named interleukin-7 (IL-7) at the 6th International Lymphokine Conference, France (2). Subsequently, it has been confirmed that IL-7 is mainly produced by thymus and bone marrow stromal cells (3), but can also be secreted by lymphoid organs (spleen, tonsil), non-lymphoid tissues (liver, lung, intestine, and skin), and tumors (colorectal cancer, prostate cancer) (4–11).

B and T lymphocytes develop from hematopoietic stem cells (HSCs) and play critical roles in regulating immune responses. IL-7 is a highly pleiotropic cytokine that is required for the efficient generation of lymphocytes from HSCs (12) and maintains the survival of B and T cells by regulating B-cell lymphoma-2 (Bcl-2) family proteins and providing proliferation signals to these lymphocytes (13–15). Gene rearrangements allow lymphocytes to recognize various antigens. For instance, IL-7 regulates the rearrangement of immunoglobulin (Ig) genes in immature B cell subsets and T cell receptor (TCR) genes in precursor T cell subsets *via* the IL-7 receptor (IL-7R) signaling pathway to ensure primary antibody repertoire and T cell diversity (16, 17). In T cells, IL-7 can also restore T cell homeostasis by enhancing thymic output (18) and trigger the expression of chemokines (CCL4, CCL25, CCL28) and integrins (α_4_β_7_, α_2_β_1_), leading to T cell homing in local organs (19–21).

IL-7 quantitatively and qualitatively modulates the responses of immune cells, such as natural killer (NK) cells, dendritic cells (DCs), B cells, and T cells, against pathogens (22–28). Indeed, IL-7 treatment promotes Th2 cell immune responses, increases the production of neutralizing antibodies, and increases the cytotoxicity of antigen-specific cytotoxic T lymphocytes (CTL) (29). Furthermore, it controls the survival of mature and memory T cells by upregulating the expression of anti-apoptotic Bcl-2 family proteins and the glycerol channel aquaporin 9, which promotes long-term immunity (30–32), as well as being an important regulator of immune balance (33). Besides, IL-7 exerts significant effects on anti-virus and anti-tumor activities, as demonstrated multiple times *in vitro* and *in vivo*. The administration of IL-7 has been found to significantly boost mouse anti-cynomolgus-monkey anti-simian immunodeficiency virus (SIV) infection (34), mouse anti-4T1 breast tumor invasion (35), lymphocytic choriomeningitis virus (LCMV) infection (36), and mouse anti-E.G7-OVA tumor invasion (37). Moreover, IL-7 has been tried to apply in patient against human diseases (**Table 1**).

**Table 1:** Application and therapeutic effect of rhIL-7 in human patient.

Disease	Results
Refractory malignancy (38)	Rejuvenating circulating T-cell profile; increasing pre-B cells proliferation and maturation; increasing circulating transitional B cells
HIV (39, 40)	Increasing the number of circulating T cells, predominantly of central memory T cells
Septic syndrome (41)	Restoring normal lymphocyte functions in septic patients, including improving CD4^+^ and CD8^+^ lymphocyte proliferations, IFN-γ production, STAT5 phosphorylation, and B cell lymphoma 2 induction
Idiopathic CD4 lymphopenia, ICL (42)	Increasing the number of circulating CD4^+^ and CD8^+^ T cells and tissue-resident CD3 T cells in the gut mucosa and bone marrow
Multidrug resistant (MDR) bacterial (43)	Increasing T cell production of IFN-γ; improving patient survival
Allogeneic hematopoietic stem cell transplantation, allo-HSCT (44)	Increasing the number and function of T cells; enhancing immune recovery
Metastatic castration-resistant prostate cancer, mCRPC (45)	Promoting the expansion of CD4^+^ and CD8^+^ T cells, and CD56^bright^ natural killer (NK) cells; improving antigen-specific humoral and T cell proliferative responses

Given the powerful biological functions of IL-7, several groups have successfully used IL-7 as a molecular adjuvant to enhance immunogenicity and prolong the protection period of vaccines against human, avian, and canine-related pathogens (46–48). In this review, we summarize and update our understanding of the biological function, mechanism, and adjuvanticity of IL-7 and highlight this cytokine as a promising molecule for future vaccine research.

## IL-7 Is Essential for Immune Cell Development

Lymphocytes such as B cells, T cells, and NK cells differentiate from common lymphoid progenitors (CLPs) derived from HSCs. CLPs differentiate into lymphocytes with the help of several cytokines, including IL-7, which plays important roles in cell differentiation, proliferation, survival, and activation. In particular, IL-7 is the predominant cytokine associated with B and T cell development, as demonstrated by Von et al. who reported that mice lacking IL-7 or IL-7Rα are unable to maintain normal numbers of B and T cells (49). NK cells were one of the earliest reported lymphocytes and are the main effector cells of the innate immune system, with essential roles in early host defenses against intracellular pathogen infection (50). In addition, IL-7 contributes towards maintaining and enhancing NK cell-based cytotoxicity (24). DCs are considered to be the most powerful antigen-presenting cells (APCs) and play central regulatory roles in immunity, such as in antigen recognition, processing, and peptide presentation to naive T cells (Tn) *via* the major histocompatibility complex (MHC), which effectively activates adaptive immune responses (51). Since IL-7 is critical for bone marrow-derived and peripheral blood monocyte-differentiated DCs (25), it may play crucial roles in host immune system development and immune response regulation. Taking these facts into account, the review next expands on the role of IL-7 on the development of immune cells.

### IL-7 in B Cell Development

Cytokine action causes CLPs to sequentially progress from pro-B cells (Igα/Igβ expressed and B cell receptor (BCR) gene rearrangement) to pre-B cells (pre-BCR complex expression) and finally develop into immature B cells (membrane IgM molecule engagement). Immature B cells then migrate into secondary lymphoid tissues, where they differentiate into mature B cells (membrane IgM and IgD expression). When mature B cells respond to antigens in peripheral lymphoid organs, they transiently form germinal centers (GCs) where Ig genes are hypermutated and selected, leading to the formation of plasma cells and memory B cells (52, 53).

Previous studies have shown that the cytokines IL-4, IL-7, IL-9, IL-13, and IL-21 play important roles in the development of B cells. In particular, IL-7 is essential for B cell development since it can enhance and maintain B lymphopoiesis-specific transcription factor early B cell factor 1 (EBF1) expression to induce B cell production and to cause early B cell differentiation at the CLP stage (54, 55). IL-7 also critically assists the transformation of pre-pro-B cells into pro-B cells, with pre-pro-B cells from IL-7^-/-^ mice losing their ability to differentiate into pro-B cells (56). In this process, pre-pro-B cell growth stimulating factor (PPBSF) is an important cofactor of IL-7, which up-regulates the expression of IL-7Rα, making pro-B cells better respond to IL-7 (57). In addition, IL-7 is essential for pre-B cell formation from pro-B cells in the bone marrow: when IL-7 is neutralized using a monoclonal antibody, pro-B cells are unable to develop into pre-B cells (58), but their differentiation is rescued by IL-7 supplementation (59). However, the function of IL-7 varies between different early B cell subsets. For instance, recombinant IL-7 administration was found to increase the number of pro-B and pre-B cells in wildtype mice and renal adenocarcinoma mice through transducing replicative signals for cell proliferation by inducing signal transducer and activator of transcription-5 (STAT5) phosphorylation (60–62), whereas other experiments have shown that it provides survival signals instead of proliferation signals for CD19^-^ B-cell progenitors (63).

**Figure 1:**
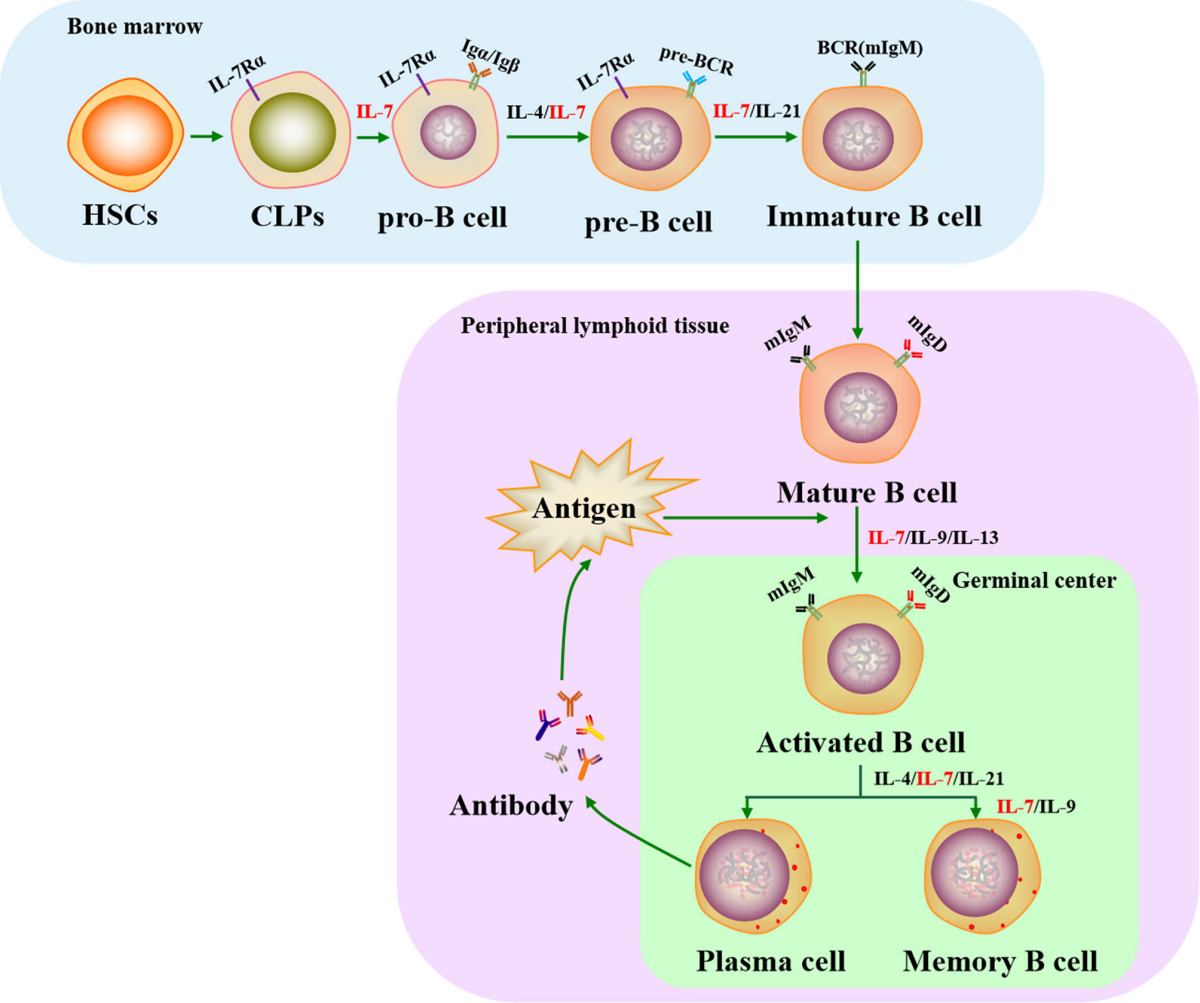
Role of IL-7 in the life cycle of B cells. IL-7 plays an essential role in B cell development in both bone marrow and peripheral lymphoid tissues. It is involved in regulating the production of pro-B, pre-B, immature B, plasma, and memory B cells. HSCs, hematopoietic stem cells; CLPs, common lymphoid progenitors; IL-7Rα, IL-7 receptor α chain; BCR, B cell receptor; mIgM, membrane IgM; mIgD, membrane IgD.

The Ig loci of B cells recombine in a stepwise and reproducible manner. Ig heavy chain (HC) genes are rearranged in pro-B cells, whereas Ig light chain (LC) genes are rearranged in pre-B cells (64). IL-7 is essential for orchestrating the rearrangement of both the Ig HC and LC loci in early B cell development (65). In addition, IL-7 co-ordinates with the transcription factor paired box protein 5 (PAX5) to regulate Ig HC variable-region (VH) gene rearrangement that generates antibody repertoire during B lymphocyte development (66). In particular, IL-7 influences the distal VH locus by activating H3K36me2 histone modification, whereas PAX5 controls the proximal VH locus by increasing H3K27me3 histone modification (65). In IL-7^-/-^ mice, splenic B cell structure is absent or aberrant and the IgM xenoantibody (IgMXAb)-producing function of marginal zone B cells is blocked (67). During B cell development, IL-7 regulates the expression of anti-apoptotic genes (Bcl-2, Bcl-xL, Mcl-1) and pro-apoptotic genes (Bax, Bad, Bim) to affect the survival of precursor B cell subsets *via* IL-7R-mediated signals (68, 69). A previous study showed that mature B cells accumulate in IL-7^-/-^ mice, indicating that immature B cells do not require IL-7 for survival or further development once they have left the bone marrow (69). Although the conclusions of recent studies have been controversial, IL-7 has been harnessed as a molecular adjuvant in vaccines to significantly promote the production of neutralizing antibodies and GC B cells (70, 71), suggesting that IL-7 may assist mature B cell development in the formation of plasma and memory B cells. This function may be indirectly regulated by IL-7 through T follicular helper (Tfh) cells, which assist B cells to produce pathogen neutralizing antibodies and to form memory B cells (72, 73). They prove that IL-7 plays a directly or indirectly role in the development of B cells (**Figure 1**). Although IL-7 is a key cytokine in B cell progenitors development in the mouse, it has less critical function in human (74).

### IL-7 in T Cell Development

T cells play a key role in cellular immunity against intracellular pathogens. Pro-T cells differentiate and mature from HSCs that have migrated into the thymus, a primary lymphoid organ, where they transition through several developmental stages, including double-negative T cells (DN, pre-T cells), double positive T cells (DP, immature T cells), and single positive T cells (SP, Tn cells). Tn cells migrate into secondary lymphoid tissues where they can encounter antigens, leading to their activation, proliferation, and differentiation into effector T cells. Approximately 90–95% of effector T cells die after pathogen clearance and the remaining cells become memory T cells (75, 76).

**Figure 2:**
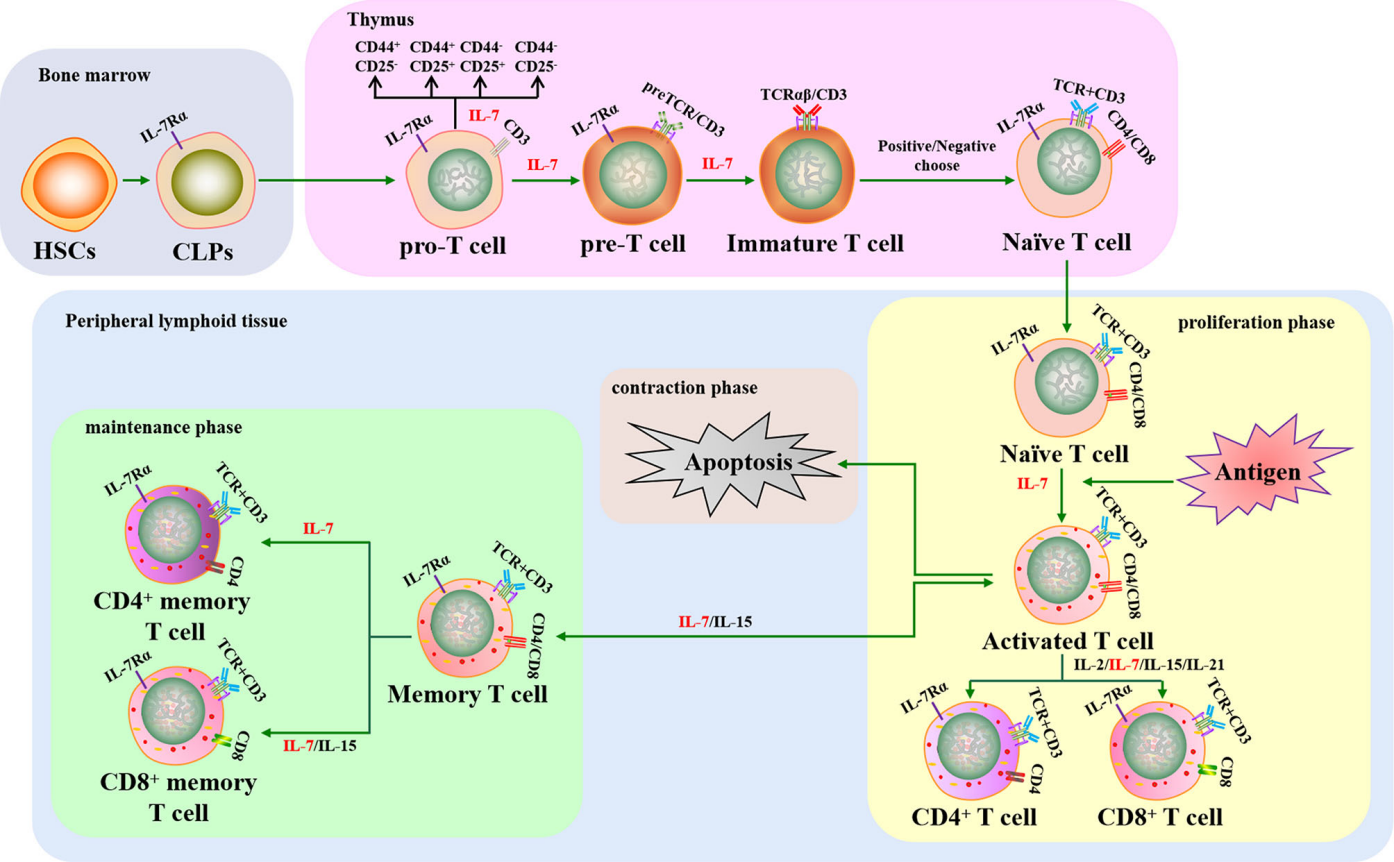
The crucial role of IL-7 in T cell development and activation. IL-7 contributes to T cell lymphopoiesis and survival. When T cells encounter antigens, IL-7 can boost T cell immune responses to fight against pathogens, including rescuing T cells from apoptosis, promoting naive T cells to differentiate into effector T cells, and improving memory T cell production. HSCs, hematopoietic stem cells; CLPs, common lymphoid progenitors; IL-7Rα, IL-7 receptor α chain; TCR, T cell receptor.

Previous studies have shown that T cell development is regulated by several cytokines, including IL-2, IL-7, IL-15, and IL-21. As a pluripotent cytokine, IL-7 is important for regulating T cell survival, proliferation, differentiation, and activation (**Figure 2**). In addition, IL-7^-/-^ mice display approximately 10-20-fold fewer total T cells, indicating that IL-7 plays a critical role in T cell development (77). However, the demand for IL-7 in T cell development varies at different stages and the dynamic interplay between IL-7 requirement and the T cell subset is regulated by IL-7R. Pro-T, pre-T, Tn, and memory T cell subsets display relatively high IL-7R expression, whereas IL-7R expression is low or absent in immature T and activated T cell subsets. Therefore, the effect of intrinsic IL-7 may be greater during the pro-T, pre-T, Tn, and memory T cell stages than in immature and activated T cells (78). Overall, several studies have assisted us to understand the important function of IL-7 in the development and activation of T cells *in vitro* and *in vivo*.

Thymocytes in IL-7^-/-^ mice have been shown to arrest in G0/G1 phase and lose Bcl-2 expression, leading to defects in T lymphopoiesis that can be rescued when the thymocytes are cultured with IL-7 (79). Subsequent studies have demonstrated that IL-7 contributes towards T lymphopoiesis by enhancing the expression of Bcl-2, Cdk2, Rb, GSK-3, and Mcl-1 and downregulating p27, Bax, Bad, and Bim (80–85). IL-7 can also rescue thymic CD4^+^CD8^+^ T cell subsets from apoptosis and is required to sustain the metabolism of Tn cells to inhibit cell atrophy (86). Long-term IL-7 monoclonal antibody treatment was found to interrupt the proliferation of CD44^+^CD25^+^ T cells in mice, causing the sharp depletion of the CD4^+^CD8^+^ T cell subset (87). In addition, studies have shown that IL-7 tightly regulates Tn cell proliferation to maintain T cell homeostasis in peripheral lymphoid tissues (33, 35, 88). However, the role of IL-7 in early T cell differentiation *in vitro* remains controversial. One study showed that IL-7 crucially controls the development of CD4^-^CD8^-^ DN T cells into CD4^+^CD8^+^ DP T cells and the differentiation of DP T cells into CD4^+^CD8^-^/CD4^-^CD8^+^ SP T cells when HSCs were cultured *in vitro* (89). Conversely, the transition of DN T cells into DP T cells was reduced when CD4^-^CD8^-^ DN thymocytes were cultured with IL-7 *in vitro*, indicating that IL-7 mainly provides survival signaling rather than DN T cell subset differentiation signaling (90). Later research demonstrated that IL-7 signaling was required for DN-DP differentiation, but it could delay DN T cells differentiation into DP T cells for maintaining DN self-renewal *in vitro* and *in vivo* (91). Additionally, IL-7 could induce the expression of CXCR4, a chemokine receptor, to support CD4^+^ and CD8^+^ SP T cell survival and recruitment in secondary lymphoid tissues (92, 93). Therefore, IL-7 is a crucial cytokine in regulating T cell production, survival, proliferation, and differentiation.

IL-7 can also boost T cell immune responses against pathogens by promoting the differentiation of Tn cells into effector T cells and then memory T cells (18, 94, 95). IL-7 has been used as an activator to augment mature T cell immune responses against chronic viruses and cancers. Recent reports have shown that IL-7 can stimulate CD8^+^ T cell proliferation and promote their cytotoxicity by upregulating perforin, granzyme B, and IFN-γ expression in patients with viral infection and tumors (96–99). The cytokine promotes the activation of both CD4^+^ and CD8^+^ T cells by increasing CXCR3 expression to control Lewis lung tumor growth (100). Ubiquitin ligases are crucial factors in protein degradation that also participate in both the innate and adaptive immune responses (101) and are involved in IL-7-mediated T cell immune responses. This notion is supported by the fact that IL-7 represses Casitas B lymphoma-b (Cbl-b) expression and enhances SMAD-specific E3 ubiquitin protein ligase-2 (Smurf2) expression to augment T cell activation during antitumor immune responses (102). Increased IL-7 usage can also enhance pathogen-specific memory T cell responses against cancer and chronic viral infections (32, 103). In addition, IL-7 serves as a key factor in promoting T-cell reconstitution, with IL-7 treatment increasing CD4^+^ T-cell circulation to maintain CD4^+^ T cell homeostasis in idiopathic CD4 lymphopenia (ICL) and human immunodeficiency virus (HIV) infections (104–106). Furthermore, it has been confirmed that the homeostatic proliferation and survival of memory CD4^+^ T cells rely on IL-7, whereas these aspects of memory CD8^+^ T cells require a combination of IL-7 and IL-15 (12, 107–110). Hence, IL-7 can enhance cell-mediated immune responses, including antigen-specific and -non-specific cellular immune responses, by promoting the production and function of effector T cells and memory T cells.

### IL-7 in NK Cell and DC Development

NK cells are a fundamental component of bodily frontline defense systems that participate in both innate and adaptive immune responses against tumors and viruses (111). IL-7Rα is expressed in thymus-dependent and -independent NK cell progenitors (NKPs) in mouse lymph nodes, indicating that IL-7 signaling may contribute towards their development (112). IL-7 not only plays important roles in NK cell maturation, but also in their survival *via* the anti-apoptotic protein Bcl-2 (113). In addition, IL-7 is strictly responsible for maintaining NK cell homeostasis (114) and is related to their activation. Dadmarz et al. showed that IL-7 could stimulate the generation of lymphokine-activated killer cells from mouse CD56^bright^ NK cells and enhance the cytotoxicity of mature NK cells (115). These findings are consistent with the ability of IL-7 to promote the cytolytic activity of mature CD56^bright^ NK cells against K562 cells by increasing CD69 expression, IFN-γ production, and CD107α expression (116). However, compared with IL-2, IL-15 and IL-21, IL-7 plays a minor role in initiating and sustaining the proliferation of NK cell (117).

DCs are a major type of antigen-presenting cell derived from bone marrow that recognize, process, and present antigens to T cells and then induce T cell-dependent immune responses. Mature DCs further differentiate into conventional DCs (cDCs) and plasmacytoid DCs (pDCs), which regulate the activation of adaptive immunity (118, 119). When DC progenitors isolated from the thymus were cultured together with GM-CSF, IL-3, and IL-7 *in vitro*, efficient DC differentiation was observed compared to stimulation with GM-CSF alone (120), suggesting that IL-7 is involved in DC differentiation. Moreover, DCs derived from both bone marrow and lymph nodes express IL-7R, while reduced proportions of cDCs and pDCs have been observed without IL-7 (25). Thus, it has been clarified that IL-7 participates in DC development.

Taken together, IL-7 is an essential cytokine in regulating both innate and adaptive immune responses through participation in B cell, T cell, NK cell and DC development to fight against pathogen infection. Although some controversial issues regarding immune cells development require further study, the feature of IL-7 lays the foundation for its application in vaccine research.

## IL-7 Signaling Pathway

**Figure 3:**
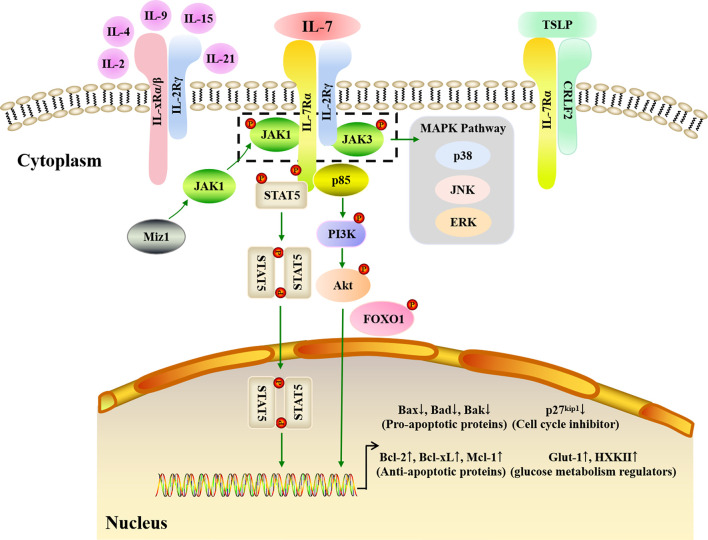
IL-7 signaling pathways. IL-7 must combine with the IL-7R to carry out its biological function. IL-7R is a transmembrane heterodimer consisting of the α chain and γ chain (common γ chain, γc chain). IL-7Rα is used by IL-7R and the thymic stromal lymphopoietin receptor (TSLPR), while γc is shared by IL-7R, IL-2R, IL-4R, IL-9R, IL-15R, and IL-21R. JAK-STAT, PI3K-Akt, and MAPK pathways are involved in IL-7 signaling transduction. When IL-7 binds to IL-7Rα, the α and γc chains dimerize. JAK1 and JAK3 are major kinases that respond to IL-7/IL-7R signaling in the JAK-STAT pathway. Myc interacting zinc finger protein 1 (Miz1) recruits JAK1 to IL-7Rα. The phosphorylation of JAK1 and JAK3 triggers STAT5 phosphorylation and dimerization to upregulate anti-apoptotic gene expression and downregulate pro-apoptotic gene expression. In addition, the MAPK pathway is also activated by the phosphorylated JAK1 and JAK3. When p85 tethers to IL-7Rα, the PI3K-Akt pathway is activated. Subsequently, the Akt is phosphorylated, which then induces glucose metabolism regulator gene expression, and inhibits p27 kinase inhibitor protein 1 (p27kip1) expression with phosphorylated Forkhead box protein 1 (FOXO1).

The biological effects of IL-7 are mediated by binding to its receptor, IL-7R, which is a transmembrane heterodimer of the IL-7Rα (CD127) and γ (CD132) chains. The α chain is used by IL-7R and thymic stromal lymphopoietin receptor (TSLPR), also termed cytokine receptor-like factor 2 (CRLF2), while the γ chain (or common γ (γc) chain) is shared by IL-7R, IL-2R, IL-4R, IL-9R, IL-15R, and IL-21R (121). IL-7Rα is mainly found in T, B, and NK cells as well as DCs, innate lymphoid cells (ILCs), and lymphoid tissue inducer (Lti) cells (122), whereas γc is expressed in all HSC-derived cell types (84). Both IL-7Rα and γc are high-affinity IL-7 receptors; however, non-receptor kinases and adaptors are employed to transduce IL-7 signals *via* IL-7R since IL-7Rα and γc both lack intrinsic tyrosine kinase activity. When IL-7 binds to IL-7Rα, the α and γc chains dimerize, triggering kinase activation (**Figure 3**). Subsequent IL-7/IL-7R signal transduction is mainly dependent on the Janus kinase (JAK)-STAT and phosphatidylinositol 3-kinase (PI3K)-Akt pathways.

The JAK family consists of JAK1, JAK2, JAK3, and tyrosine kinase-2 (TYK-2), while the STAT family has seven members: STAT1, STAT2, STAT3, STAT4, STAT5α, STAT5β, and STAT6. JAK1, JAK3, and STAT5 are the predominant proteins that respond to IL-7/IL-7R signaling: when IL-7 combines with IL-7R, JAK1 and JAK3 tether to IL-7Rα and γc, respectively, alongside IL-7Rα C-terminal phosphorylation. Next, STAT1, STAT3, and STAT5 are recruited and phosphorylated by JAK1 and JAK3 (123), with phosphorylated STAT5 production occurring in an IL-7 dose-dependent manner (124). Following its phosphorylation, STAT5 dimerizes and is translocated into the nucleus, which in turn triggers the expression of downstream genes involved in cell survival and proliferation (125–127). *Via* the JAK-STAT pathway, IL-7 upregulates the expression of anti-apoptotic genes mainly belonging to the Bcl-2 family, including Bcl-2, Mcl-1, and Bcl-xL, while also downregulating pro-apoptotic genes, including Bax, Bad, and Bak, to promote the survival of cells *in vitro* and *in vivo* (128). Lu et al. quantified B cell apoptosis and examined the expression of Bcl-2 family proteins in IL-7^-/-^ mice and IL-7 transgenic mice, finding that IL-7 strongly increased Bcl-2 levels and decreased Bax levels to promote precursor B cell survival (128). Myc interacting zinc finger protein 1 (Miz1) is a crucial activator that also participates in the JAK-STAT signaling transduction pathway by recruiting JAK1, activating STAT5, and upregulating Bcl-2 expression when IL-7 triggers early B and T cell survival and proliferation (129, 130). Therefore, the lymphocyte survival signaling provided by IL-7 is predominantly dependent on the JAK-STAT pathway.

The PI3K-Akt signaling transduction pathway is an important intracellular signaling pathway that regulates cell growth, survival, and glucose metabolism (131). Indeed, PI3K inhibitors have been found to attenuate the proliferation and activation of murine T cells in response to IL-7 (132), while Akt phosphorylation increased when IL-7 was used to improve adipose-derived stem cell differentiation (133). The pathway starts with the recruitment of p85, a regulatory subunit of PI3K, when IL-7 interacts with IL-7R. P85 then associates with IL-7Rα and induces the phosphorylation of tyrosine in the IL-7Rα cytoplasmic tail (134). Subsequently, Akt is activated to induce downstream gene expression (135). p27 kinase inhibitor protein 1 (p27^kip1^) is a crucial molecule in this pathway and its expression is downregulated by PI3K-Akt signaling *via* Forkhead box protein 1 (FOXO1) phosphorylation (136). p27^kip1^ degradation can induce cyclin D1 expression and promote the G1 to S phase transition for cell proliferation during IL-7-dependent cell cycle progression (80, 137). Furthermore, PI3K-Akt signaling can drive the cytoplasmic localization of p27^kip1^ and thereby allow cell proliferation (138, 139). Glucose is the main nutrient for cell development and activation, and studies have shown that IL-7 can upregulate the expression of the glycolytic enzymes hexokinase II (HXK II) and glucose transporter-1 (Glut-1), thereby augmenting glycolysis by increasing glucose uptake (140, 141). Furthermore, it has been clarified that IL-7-mediated glucose utilization occurs in a PI3K-Akt-dependent fashion (83).These results suggest that the PI3K-Akt pathway is necessary for effective IL-7 signaling during cell cycle progression.

In addition to the JAK-STAT and PI3K-Akt pathways, mitogen-activated protein kinases (MAPK), including p38 kinase, extracellular signal regulated kinase (ERK), and c-Jun N-terminal kinase (JNK), may also contribute towards IL-7/IL-7R signaling. Importantly, the MAPK pathway allows the transduction of signals from the cell membrane to the nucleus. The p38 MAPK pathway may play a critical role in IL-7 signaling since specific p38 inhibitors can suppress T cell proliferation driven by IL-7 (142). Moreover, IL-7 withdrawal results in IL-7-dependent thymocyte death caused by blocking the activation of p38 and JNK kinases (143). In addition to JAK3 activation, the phosphorylation of p44, an ERK isoform, was found to significantly increase after IL-7 stimulation in murine T helper cells (144). Furthermore, IL-7 signaling may affect the migration of peripheral blood monocytes in rheumatoid arthritis (RA), a chronic autoimmune disorder caused by ERK (145). Therefore, the ERK MAPK pathway appears to play a role in IL-7-mediated signal transduction to regulate cell development and activation.

## IL-7 Applications in Vaccines

An ideal vaccine would permanently protect the host from related pathogens after only one immunization; therefore, it is important to develop efficient vaccines with broad, robust, and long-lasting immunogenicity. Cytokines are commonly employed as adjuvants to enhance and extend the vaccine effect, with early studies revealing that IL-7 can improve vaccine protection by driving T lymphocyte responses as a molecular adjuvant (29, 146, 147). Recent studies have also shown that IL-7 can not only enhance cellular immune responses, but also improves the humoral immune responses induced by vaccines (71, 148–150). As an adjuvant, IL-7 has been shown to stimulate antigen-specific CD4^+^ T cell, CD8^+^ T cell, and B cell responses to boost vaccine potency, as well as increasing the production of memory T and B cells to prolong the protective effects of vaccines (108, 151–154) (**Table 2**).

**Table 2:** Application and advantages of IL-7 as a biological adjuvant in vaccines.

Application	Formation	Dosage of IL-7	Delivery of IL-7	Animal	Advantage
Influenza virus (IV) (70)	rhIL-7 protein + trivalent IV inactivated vaccine	1.8 μg	i.m	Mouse	Facilitating the generation of GC B cells;Increasing neutralizing antibodies against homologous and heterologous influenza viruses
The man antigen HY (151)	rhIL-7 protein + HY antigen	5 μg/day, 27 days	i.p	Mouse	Augmenting HY specific-CD8^+^ effector T cell generation;Improving the survival of HY specific-CD8^+^ memory T cells
Lymphocytic choriomeningitis virus, LCMV (155)	rhIL-7 protein + LCMV DNA vaccine	5 μg/day, 7 days	i.p	Mouse	Increasing the number of specific mouse anti-LCMV memory CD8^+^ T cells;Enhancing T cell proliferation and cytotoxicity to improve viral clearance
Infectious bursal disease virus, IBDV (154, 156)	chIL-7 protein + IBDV inactivated vaccine	200 μg/time, 3 times, at 1 week interval	i.m	Chicken	Increasing chicken anti-IBDV antibody titers; promoting lymphocyte proliferation; up-regulating IFN-γ and IL-4 production
	ch*IL-7* gene inserted into IBDV VP2_366_ DNA vaccine	200 μg/time, 3 times, at 1 week interval	i.m	Chicken	Increasing the levels of neutralizing antibodies, IFN-γ and IL-4
Eimeria tenella-1, EF-1 (157)	chIL-7 protein + EF-1 DNA vaccine	20 μg/time, 2 times, at 1 week interval	i.m	Chicken	Boosting humoral and cellular immunity against live *Eimeria tenella* challenge
Hepatitis C virus, HCV (158)	hIL-7 plasmid + HCV DNA vaccine	200 μg/time, 6 times, at 1 month interval	Electroporation	Monkey	Increasing anti-HCV antibody levels and the T cell response
Human papillomavirus, HPV (159)	hIL-7 protein + HPV DNA vaccine	1 mg/kg	i.v	Mouse	Enhancing genital mucosal CD8^+^ T cell immune responses; promoting anti-tumor activity
Newcastle disease virus, NDV (160)	m*IL-7* gene inserted into nonlytic NDV genome	10^6^ cell/time, 2 times, at 1 week interval	s.c	Mouse	Greater levels of tumor-infiltrating CD4^+^ and CD8^+^ T cells;stronger cytotoxicity of tumor-specific CD8^+^ T cells
Rabies virus (150)	m*IL-7* gene inserted into a recombinant attenuated rabies virus genome (rRABV)	10^6^ FFU rRABV	i.m	Mouse	Increasing antigen-specific memory B cells; prolonging neutralizing antibodies production
*Mycobacterium bovis* (161)	m*IL-7* gene inserted into recombinant *M. bovis* BCG genome (rBCG)	10^6^ CFU rBCG	i.v	Mouse	Increasing the pool size of IL-17A^+^ γδ T cells; augmenting Th1 response
*Mycobacterium tuberculosis* (162)	mIL-7/mIL-15 proteins + bacille Calmette-Guerin (BCG) vaccine	500 ng of each cytokine/time, 2 times, at 3 weeks interval	i.p	Mouse	Augmenting T cell proliferation and cytokines IL-2, IFNγ production; promoting both CD4^+^ and CD8^+^ memory T cell responses
*Toxoplasma gondii* (149)	mIL-7/mIL-15 co-expression plasmids + *Toxoplasma gondii* calcium-dependent protein kinase 1 (TgCDPK1) DNA vaccine	100 μg/time, 3 times, at 2 weeks interval	i.m	Mouse	Enhancing levels of *Toxoplasma*-specific IgG2a, CD8^+^/CD4^+^ T cell frequencies

FFU, focus-forming units; CFU, colony forming unit; i.p, intraperitoneal injection; i.m, intramuscular injection; i.v, intravaginal injection; s.c, subcutaneous injection.

The IL-7 protein possesses strong adjuvant activity to improve and extend vaccine protection. Indeed, costimulation of the male HY antigen with recombinant human IL-7 (hIL-7) protein may augment HY specific-CD8^+^ effector T cell generation and improve the survival of HY specific-CD8^+^ memory T cells (155). In addition, administering recombinant hIL-7 protein with a trivalent inactivated influenza vaccine (TIV; H1N1 A/New Caledonia/20/99, H3N2 A/Fujian/411/2002, and B/Shanghai/361/2002) could facilitate the generation of GC B cells and increase neutralizing antibodies against homologous and heterologous (PR8/H1N1) influenza viruses (70). Nanjappa et al. found that the codelivery of hIL-7 protein with a DNA vaccine encoding LCMV nucleoprotein (NP) in mice during the contraction phase of the T cell response could increase the number of specific mouse anti-LCMV memory CD8^+^ T cells and enhance T cell proliferation and cytotoxicity to improve viral control (155). Using hIL-7 protein as an adjuvant may also improve mouse anti-tumor immune responses by increasing pro-inflammatory cytokine production [IL-6, IL-1α, IL-1β, IL-12, TNF-α, C-C chemokine ligand-5 (CCL-5), macrophage inflammatory protein 1α (MIP-1α)], augmenting Th17 cell differentiation, repressing Cbl-b expression, and enhancing SMAD-specific E3 ubiquitin protein ligase 2 (Smurf2) expression (98). Furthermore, recombinant chicken IL-7 (chIL-7) protein expressed in *E. coli* and administered with the inactivated infectious bursal disease virus (IBDV) vaccine was found to significantly upregulate chicken anti-IBDV antibody titers, lymphocyte proliferation, and IFN-γ and IL-4 production (163). This increased the survival rate of chickens to 91–97% compared to 78% when vaccinated with the inactivated IBDV vaccine alone (163). Alfredo et al. also assessed the adjuvanticity of chIL-7 protein with the *Eimeria tenella* elongation factor 1α (EF-1α) DNA vaccine, finding that chIL-7 boosted humoral and cellular immunity against live *E. acervulina* challenge in broiler chickens that received the EF-1α DNA vaccine (157).

Flexner et al. were the first to report the use of a cytokine gene as a biological adjuvant, having inserted m*Il-2* into the vaccinia virus genome to construct a recombinant virus as a novel vaccine. They found that m*Il-2* was able to improve the safety and immunogenicity of the vaccinia virus (164), providing a foundation for the application of cytokine genes in vaccines. Subsequently, numerous studies have attempted to co-administer cytokine genes with vaccines to improve their efficiency. IL-7 has been confirmed to exhibit efficient adjuvanticity to boost vaccine immunogenicity, with the codelivery of hIL-7-encoding plasmids with hepatitis C virus (HCV) NS2-E1E2-NS3-NS4 DNA vaccine increasing anti-HCV antibody levels and widening the T cell response compared to the DNA vaccine alone (158). A DNA vaccine co-expressing IBDV VP2_366_ and chIL-7 *via* an internal ribosome entry site (IRES) connection was constructed and then used to intramuscularly immunize SPF chickens, with significantly strengthened immunogenicity compared to the IBDV VP2_366_ DNA vaccine (156). In particular, the VP2_366_-chIL-7 DNA vaccine increased the titer of neutralizing antibodies as well as IFN-γ and IL-4 production, while chIL-7 enhanced IBDV VP2_366_ DNA vaccine protection by approximately 25% (156). The murine *Il-7* (m*Il-7*) gene has also been inserted into the genome of the nonlytic Newcastle disease virus (NDV) LX strain, which is an autologous tumor vaccine modified using the reverse genetic method that has been used against murine tumors (HCC, lymphoma, and melanoma) (160). Interestingly, the NDV LX-mIL-7 vaccine displayed strong anti-tumor activity, with mIL-7 expression by the NDV LX strain inducing higher IFN-γ production, greater levels of tumor-infiltrating CD4^+^ and CD8^+^ T cells, and stronger cytotoxicity of tumor-specific CD8^+^ T cells than the NDV LX vaccine (160). Similarly, when m*Il-7* was inserted into the recombinant canine distemper virus (rCDV) genome, it significantly promoted the generation of GC B cells and plasma cells in draining lymph nodes (LNs) (71). The number of antigen-specific memory B cells was also highly increased and the presence of virus-neutralizing antibodies was prolonged (up to 360 days) when mice were injected with a recombinant attenuated rabies virus (rLBNSE) expressing mIL-7 (150). These results suggest that the *Il-7* gene can be directly used in vaccine development. Although IL-7 has strong adjuvanticity, its immunomodulatory properties may be different in certain diseases. The vaccine efficacy of Friend Virus was no significant changed when with or without adenoviral vectors encoding hIL-7 (165). Therefore, further research is needed to pinpoint the benefits of IL-7 in different pathogen vaccines, and the adjuvanticity of IL-7 in different vector-based vaccines.

Another strategy to improve the immunogenicity of vaccines is to combine IL-7 with other cytokines. When m*IL-7* or m*IL-33* was subcloned into pVAX1 vector, both mIL-7 and mIL-33 could enhance the immunogenicity of varicella-zoster virus (VZV) glycoprotein E (gE) DNA vaccine. However, compared with the coadministration of mIL-7 and mIL-33, the DNA vaccine with mIL-7 alone enhanced the weaker VZV-specific T cell immune responses and protection (153). Coadministering mice with bacille Calmette-Guerin (BCG) vaccine and mIL-7/mIL-15 proteins promoted T cell proliferation, cytokine production, and both CD4^+^ and CD8^+^ memory T cell responses compared to the BCG vaccine alone (162), indicating that mIL-7 and mIL-15 proteins can induce long-lasting T cell immune responses against *Mycobacterium tuberculosis.* Similarly, recombinant NDV expressing mIL-7/mIL-15 also improved the anti-tumor activity of the NDV LX strain (166), while the delivery of mIL-7/mIL-15 co-expression plasmids as molecular adjuvants with the *Toxoplasma gondii* calcium-dependent protein kinase 1 (TgCDPK1) DNA vaccine enhanced *Toxoplasma*-specific IgG2a levels and CD8^+^/CD4^+^IFN-γ^+^ T cell frequencies induced by the vaccine and prolonged the survival of mice post lethal infection by approximately 7 days (149). Furthermore, m*Il-7*/m*Il-15* genes subcloned into an adenovirus were able to extend the protective efficacy of the *M. tuberculosis* subunit vaccine (LT70 and MH fusion protein) by augmenting *M. tuberculosis*-specific central memory-like T cell responses (167). When m*Il-7*/m*Il-2* were co-administered with the ovalbumin (OVA) DNA vaccine, mIL-7 and mIL-2 mutually improved its immunogenicity by up-regulating the specific antibody titer, IFN-γ production, and T cell proliferation (168). This may have occurred *via* a mechanism wherein mIL-2 induced the generation of mIL-7Rα-expressing lymphocytes, which were increased in number by mIL-7 (168). These findings show that other adjuvants are needed to enhance the adjuvanticity of IL-7 in some vaccines. As a result, further studies about the form and mechanism of IL-7 in the development of specific vaccines should be conducted.

The mucosal immune system plays a fundamental role in defending against infection through food, water, pathogens, and direct contact with mucosal surfaces. The Fc fragment is always employed to enhance the mucosal delivery of a target protein. When mice were intranasally treated with Fc-fused mouse IL-7 (mIL-7-mFc) not native IL-7 protein before lethal influenza A virus (IAV) infection, the percentage of mice that survived was significantly increased (169). In particular, mIL-7-mFc recruited both T cells and pDCs from circulation into the lungs, with T cells transitioning into lung-specific memory-like T cells (T_RM_-like) and pDCs strengthening lung-specific anti-IAV CTL responses (169, 170), thereby providing long-lasting immune responses against lethal AIV infection by altering the pulmonary immune environment. Since IL-7 is locally produced by intestinal epithelial cells, particularly epithelial goblet cells, it can regulate the phenotype and function of intraepithelial lymphocytes (IELs) and lamina propria lymphocytes (LPLs) (171, 172). Indeed, mIL-7 prevented approximately 50% of IELs from undergoing spontaneous apoptosis *via* both caspase-dependent and Bcl-2-dependent pathways *in vitro* (173). Exogenous mIL-7 was also able to reverse the mucosal damage induced by parenteral nutrition (PN) and increase the levels of secretory IgA in the intestine and bronchoalveolae, meaning that mice with PN could resist lethal *Pseudomonas aeruginosa* infection (174). In addition, the hIL-7-hFc protein not hIL-7 was shown to enhance genital mucosal CD8^+^ T cell immune responses to the human papillomavirus (HPV) GX188 DNA vaccine and promote the anti-tumor activity of the vaccine (159). The vaccine has entered preclinical trials and demonstrated that hIL-7-hFc can be used with safety (175). Together, these studies indicate that IL-7 could be used as a promising molecular adjuvant to improve mucosal immune responses to vaccines. Moreover, the formation of IL-7 with Fc fragment should be considered in future mucosal vaccine development.

## Conclusion

Vaccines play fundamental roles in helping the body to resist pathogenic infections; however, antigens alone are unable to stimulate persistent protective immune responses. To solve this problem, cytokines have been employed to improve vaccine-specific immunity and promote vaccine-specific memory immune responses. In the past three decades, numerous trials have confirmed that IL-7 can alter immune responses and promote immune reconstitution by inducing the development and activation of B cells, T cells, NK cells, and DCs. Moreover, IL-7 exhibits adjuvant properties, enhancing effector and memory cell immune responses, and has been used in preventive and therapeutic vaccines.

As mucosal vaccines always present poor immunogenicity, an appropriate adjuvant to enhance mucosal immunity is necessary. In view of the biological activity and tissue distribution of IL-7, it has been used in vaccines to enhance mucosal immune responses. It has been proved that IL-7 can not only promote the humoral and cellular immune responses of vaccines, but also improve their mucosal immunity. It can recruit DCs, NK cells, B cells, and T cells to infiltrate in local mucosal tissues. IL-7 has been employed in the development of IAV (169), HPV (159), DT (176), and other mucosal vaccines. This strategy has been demonstrated to be toxicity-free.

These findings have made IL-7 an important adjuvant in vaccine development. Here, we reviewed the biological function and mechanism of IL-7, and summarized its applications in vaccines to provide a new perspective on clinical vaccine design.

## Author Contributions

JH and ZL wrote the manuscript. RJ, MW, DZ, ML, SC, XZ, QY, YW, SZ, BT, SM, XO, DS, QG, and AC revised the review. All authors contributed to the article and approved the submitted version.

## Funding

This work was supported by the National Natural Science Foundation of China (grant number 31902286), the Program Sichuan Veterinary Medicine and Drug Innovation Group of China Agricultural Research System (SCCXTD-2021-18) and China Agriculture Research System of MOF and MARA.

## Conflict of Interest

The authors declare that the research was conducted in the absence of any commercial or financial relationships that could be construed as a potential conflict of interest.

## Publisher’s Note

All claims expressed in this article are solely those of the authors and do not necessarily represent those of their affiliated organizations, or those of the publisher, the editors and the reviewers. Any product that may be evaluated in this article, or claim that may be made by its manufacturer, is not guaranteed or endorsed by the publisher.
