# Supplementary material for: The Broad Immunomodulatory Effects of IL-7 and Its Application In Vaccines
Source: Front Immunol. 2021 Dec 10;12:680442. doi: 10.3389/fimmu.2021.680442 (PMC8702497; doi:10.3389/fimmu.2021.680442)
Supplement: Supplementary file 1 [file Table_1.docx]

Table S1 The distribution of IL-7 in human

| **organs** | **Cell type** |
| --- | --- |
| Thymus | Medullary and cortical; thymic epithelial cells |
| Lymph nodes (LNs) | Fibroblastic reticular cells; lymphatic endothelia |
| Lung | Afferent lymphatics; bronchus-associated lymphoid tissue |
| Skin | Keratinocytes; hair follicles |
| Intestine | Intestinal epithelial cells |
| Brain | Neuronal progenitor cells |
| Liver | Hepatocytes |
| Intestine | Epithelial cells |
| Bone marrow | VCAM1+ stromal cells |
| Spleen | Fibroblastic reticular cells |
